# Postoperative outcomes of kidney transplant recipients undergoing non-transplant-related elective surgery: a systematic review and meta-analysis

**DOI:** 10.1186/s12882-020-01978-4

**Published:** 2020-08-25

**Authors:** Dharmenaan Palamuthusingam, Kuhan Kunarajah, Elaine M. Pascoe, David W. Johnson, Camel M. Hawley, Magid Fahim

**Affiliations:** 1 Metro South Integrated Nephrology and Transplant Services, Logan Hospital, Armstrong Road & Loganlea Road, Meadowbrook, Queensland 4131 Australia; 2 Faculty of Medicine, University of Queensland, St Lucia, Queensland 4072 Australia; 3 School of Medicine, Griffith University, Mount Gravatt, Queensland, Australia; 4 Department of Medicine, Sunshine Coast University Hospital, Doherty St, Birtinya, Queensland 4575 Australia; 5 Centre for Health Services Research, University of Queensland, St Lucia, Queensland 4072 Australia; 6 Metro South Integrated Nephrology and Transplant Services, Princess Alexandra Hospital, 199 Ipswich Road, Woolloongabba, Queensland 4074 Australia; 7 Translational Research Institute, Brisbane, Australia

**Keywords:** Perioperative outcomes, Surgical risk, Kidney transplant, Postoperative mortality, Myocardial infarction, Stroke, Infection

## Abstract

**Background:**

Reliable estimates of the absolute and relative risks of postoperative complications in kidney transplant recipients undergoing elective surgery are needed to inform clinical practice. This systematic review and meta-analysis aimed to estimate the odds of both fatal and non-fatal postoperative outcomes in kidney transplant recipients following elective surgery compared to non-transplanted patients.

**Methods:**

Systematic searches were performed through Embase and MEDLINE databases to identify relevant studies from inception to January 2020. Risk of bias was assessed by the Newcastle Ottawa Scale and quality of evidence was summarised in accordance with GRADE methodology (grading of recommendations, assessment, development and evaluation). Random effects meta-analysis was performed to derive summary risk estimates of outcomes. Meta-regression and sensitivity analyses were performed to explore heterogeneity.

**Results:**

Fourteen studies involving 14,427 kidney transplant patients were eligible for inclusion. Kidney transplant recipients had increased odds of postoperative mortality; cardiac surgery (OR 2.2, 95%CI 1.9–2.5), general surgery (OR 2.2, 95% CI 1.3–4.0) compared to non-transplanted patients. The magnitude of the mortality odds was increased in the presence of diabetes mellitus. Acute kidney injury was the most frequently reported non-fatal complication whereby kidney transplant recipients had increased odds compared to their non-transplanted counterparts. The odds for acute kidney injury was highest following orthopaedic surgery (OR 15.3, 95% CI 3.9–59.4). However, there was no difference in the odds of stroke and pneumonia.

**Conclusion:**

Kidney transplant recipients are at increased odds for postoperative mortality and acute kidney injury following elective surgery. This review also highlights the urgent need for further studies to better inform perioperative risk assessment to assist in planning perioperative care.

## Background

Patients with end-stage kidney disease (ESKD) derive a notable improvement in survival and quality of life compared to patients who remain on chronic dialysis [1, 2]. However, kidney transplant recipients are a potentially high risk surgical population for both fatal and non-fatal cardiovascular, infectious, and wound complications, by virtue of having a higher comorbid illness burden, immunosuppression-related cardio-metabolic derangements, and an augmented immune response [3]. The evidence regarding the odds of adverse postoperative outcomes in kidney transplant recipients is conflicting, limited by small studies, and inconsistently adjust for confounding factors. For example, a previous retrospective study of 1305 kidney transplant recipients reported higher rates of in-hospital mortality following elective colorectal surgery compared to the general population, whereas another study of 70 kidney transplant recipients reported no such differences following coronary artery bypass grafting [4, 5]. Therefore, a systematic synthesis of published literature on both fatal and non-fatal outcomes of kidney transplant recipients undergoing elective surgery would help better inform shared decision making by clinicians and patients.

The aim of this study was to perform a systematic review and meta-analysis of kidney transplant recipients undergoing elective non-transplant surgery to assess their odds of both fatal and non-fatal post-operative outcomes compared to patients with non-transplanted patients.

## Methods

This systematic review adhered to the Meta-analysis of Observational Studies in Epidemiology (MOOSE) proposal for reporting [6] and Preferred Reporting Items for Systematic Reviews and Meta-analyses (PRISMA) guidelines [7], with a protocol registered in PROSPERO (CRD42019127267).

### Selection criteria and search strategy

All cohort studies comparing post-operative mortality and morbidity in adult (aged 18 years or older) kidney transplant recipients with non-transplanted patients in the general population were considered for inclusion. All types of surgery requiring a general anaesthetic were considered, including general, orthopaedic, cardiac, vascular and urological/gynaecological surgery. Kidney transplantation and related urological procedures were excluded. Studies in which more than 25% of the procedures were emergent (defined as an acute illness leading to an emergency presentation or an unplanned admission requiring a surgical procedure) were excluded because they have an inherently higher odds of perioperative complications. Studies reporting aggregate outcomes for a mixed population of solid organ transplant recipients were included if > 70% of the population comprised kidney transplant recipients.

We searched MEDLINE and Embase from inception to January 2020, without language restriction using a combination of relevant keywords including surgery, kidney transplant, postoperative, perioperative, mortality and their variants (see supplementary Table 1a and b). Exploded MeSH terms for perioperative medicine and kidney transplant recipients were also used. Full-text articles obtained were hand searched for further references. Tangential electronic exploration using links to related texts was also performed for additional materials. Case-control studies, opinion papers, case reports and editorials were excluded.

Database of Abstracts of Reviews of Effects (DARE), the Cochrane Database of Systematic Reviews (CDSR), National Institute for Health and Clinical Excellence (NICE) and the NIHR Health Technology Assessment (NIHR HTA) programme websites were all searched for existing reviews.

### Data extraction and outcome definition

Two researchers (D.P & K.K) independently reviewed all abstracts identified in the initial search to assess study eligibility. Any disagreements were resolved by a third reviewer (M.F). Type of surgery, patient numbers, summary statistics for baseline characteristics (including baseline kidney function, immunosuppression, presence of cardiovascular disease, peripheral vascular disease, diabetes mellitus, hypertension and smoking status), and frequency of post-operative outcomes in each group were extracted from full-text manuscripts of eligible studies. An individually tailored data request form was used to obtain additional data from corresponding authors, including aggregated summary data of kidney transplant recipients baseline characteristics and outcomes.

The primary outcome was all-cause mortality, defined as either 30-day mortality or death within the same hospitalisation as the index surgery. Secondary outcomes were postoperative myocardial infarction, stroke, congestive cardiac failure, pneumonia, surgical site infection (both superficial and deep), sepsis, acute kidney injury (AKI), thromboembolic events and return to theatre. Outcome definitions of complications were noted at the time of data extraction. The severity of each complication was measured using the Clavien-Dindo Classification, which was used to evaluate the implications of those postoperative complications on patients’ treatment courses and outcomes [8]. The scale ranged from 1 to 5, with Grade 1 referring to any deviation to the usual postoperative course and Grade 5 complications leading to postoperative death [9]. The methodological quality of each study was assessed using the Newcastle-Ottawa Scale (NOS) evaluating the selection of the study groups (0–4 stars), comparability of the groups (0–2 stars), and ascertainment of the outcome of interest (0–3 stars) [10].

### Statistical analysis

Publication bias was assessed using a funnel plot and Egger’s test for funnel plot asymmetry. Cohen’s kappa was calculated to assess inter-rater reliability of study selection. Heterogeneity was assessed using *I*^*2*^ using the t-statistic for degrees of freedom due to the small number of studies [11, 12].

Unadjusted odds ratios [OR] and 95% confidence intervals (CIs) were calculated for both fatal and non-fatal outcomes reported in each study to ensure consistency using the absolute number of events in each group. Adjusted OR and 95% CI were recorded from studies that performed a multivariable analysis, adjusting for age as a minimum. As pre-specified in the protocol, odds ratio estimates were calculated using the DerSimonian and Laird (DL) method of random effects meta-analysis for each surgical discipline. Further meta-analysis using the Hartung-Knapp-Sidik- Jonkman method (HKSJ) was also performed for comparison due to small number of studies [12].

Unadjusted and adjusted ORs for both primary and secondary outcomes in kidney transplant recipients versus non-transplanted patients were pooled by surgical disciplines, but not across disciplines due to differences in risks inherent to a particular discipline, potential for an interaction between surgical risk and transplant status, and inability to exclude differential bias in selection of surgical candidates. Meta-regression was performed using the random effects model to assess whether the unadjusted effect size was associated with important study level covariates, including age and pre-operative co-morbidity (ischemic heart disease and diabetes). Sensitivity analyses, excluding studies reporting aggregated outcomes involving other solid organ transplant recipients, were also conducted for all outcomes.

L’Abbé plots were used to explore studies with divergent results and evaluate potential contributions of study group characteristics to such differences [13]. Influence analysis was also performed to evaluate the influence of each study on the overall meta-analysis summary estimate and identify outlier studies that may have had an undue influence on results [14]. Six reviewers (D.P, E. P, D. J, C. H, and M.F) discussed the overall strength of evidence and graded it according to Grading of Recommendations Assessment, Development and Evaluation (GRADE) working group recommendations [15].

Statistical analysis was performed with Stata 14.0 for Windows. Statistical significance was defined as a two-sided *p*-value < 0.05.

## Results

### Study selection and characteristics

**Fig. 1:**
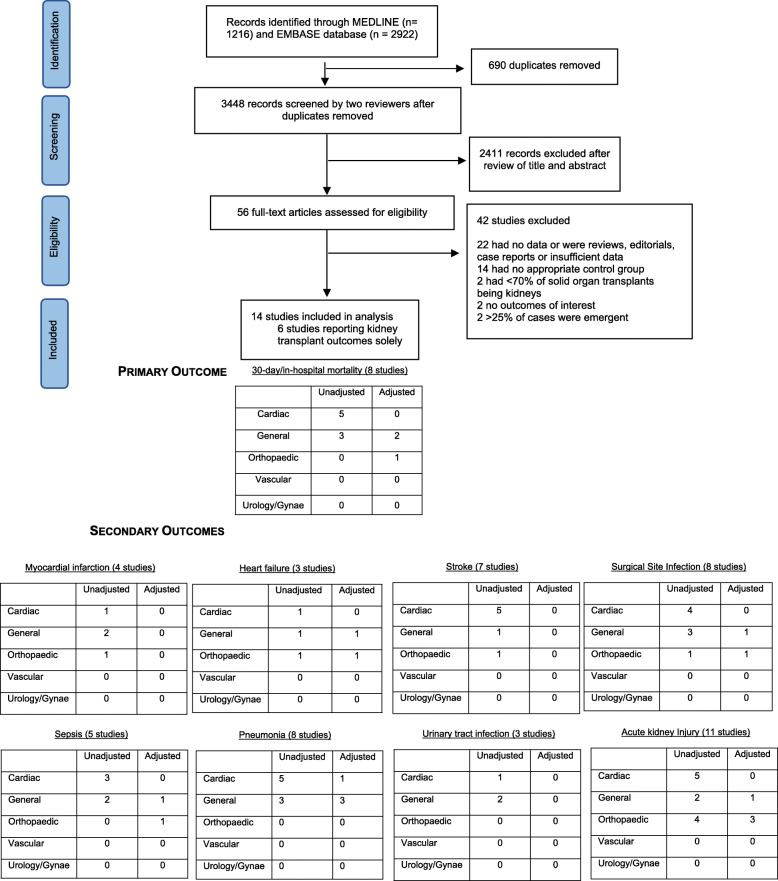
Study selection

In total, 3448 abstracts were reviewed, from which 56 full-text articles were retrieved and evaluated (See Fig. 1). Fourteen studies, involving 15,481 solid organ transplant recipients of whom 14,427 (95%) were kidney transplants, and 7,807,705 non-transplanted patients satisfied the inclusion criteria (Table 1: Summary of included studies). Non-emergent cardiac (5 studies) [5, 18, 20, 24, 27] and orthopaedic (5 studies) [16, 17, 19, 22, 23] surgery were the most commonly reported types of surgery. General surgical outcomes were reported in 3 studies [4, 21, 25]. A single study assessed outcomes following urological/gynaecological procedures [26] and there were no studies in vascular surgery. Six studies assessed a single surgical procedure [16, 17, 19, 22, 23, 26], while the remainder examined a combination of discipline-specific surgical interventions [4, 5, 18, 20, 21, 24, 25, 27]. All but 4 studies [18, 21, 22, 24] were from North America.

**Table 1:** Baseline characteristics of included studies

Author	Country	Type of Surgery	Total number of patients in study (n)		Mean age years ± SD [IQR]		Ischemic heart disease (%)		Diabetes (%)		Outcomes reported
			Non-transplanted patients	Transplant patients	Non-transplanted patients	Transplant patients	Non-transplanted patients	Transplant patients	Non-transplanted patients	Transplant patients	
Cavanaugh, 2015 [16]	USA	Orthopaedic; Joint Arthroplasty	2,583,529	3209	66.8 ± 11.6	53.5 ± 13.1	–	–	–	–	Cardiac failure, surgical site infection, PE/DVT, pneumonia, sepsis, Acute kidney injury
Choi, 2013 [17]	USA	Orthopaedic; Total hip arthroplasty	2044	222	57.4 ± 15.2	44.2 ± 11.2	110 (5.4)	12 (5.4)	233 (11.4)	26 (11.7)	Acute kidney injury
Farag, 2017 [18]	UK	Cardiac; CABG	70	70	58.6 ± 10.3	57.8 ± 11.2	32 (45.7)	28 (40.0)	29 (41.4)	42 (60.0)	30-day mortality, Surgical site infection, Pneumonia, Sepsis, Stroke, GI, Transfusion, Return to theatre, ICU admission, Acute kidney injury, Urinary tract infection
Halabi, 2013 [4]	USA	General; colorectal	1,402,020	1305	65 ± 11.0	59.0 ± 3.1	161,029 (11.5)	272 (20.8)	347,041 (24.8)	772 (59.0)	In-hospital mortality, surgical site infection, PE/DVT, pneumonia, stroke, GI, transfusions, Acute kidney injury, Urinary tract infection
John, 2007 [5]	USA	Cardiac; CABG & Valve	895	70	61 ± 13.6	52.1 ± 9.9	881 (98.4)	50 (71.4)	350 (39.1)	65 (92.9)	30-day mortality, myocardial infarction, surgical site infection, pneumonia, sepsis, stroke, GI, Return to theatre, ICU admission, Acute kidney injury
Klement, 2016 [19]	USA	Orthopaedic; Total knee arthroplasty	1,685,295	3334	69.5 ± 9.7	67 ± 8.1	–	–	–	–	Myocardial infarction, Cardiac failure, PE/DVT, stroke, Transfusions, Acute kidney injury
Kohmoto, 2018 [20]	USA	Cardiac; CABG & Valve	345	115	60 ± 14	58 ± 11	147 (42)	38 (33)	205 (59)	71 (62)	30-day mortality, Surgical site infection, Return to theatre, Pneumonia, Acute kidney injury, Stroke, 30-day readmission
Lederer, 2019 [21]	Germany	General; Abdominal surgery	84	84	59.8 ± 12.7	59.0 ± 9.0	–	–	–	–	In-hospital mortality return to theatre, surgical site infection, urinary tract infection, pneumonia, transfusions, pulmonary embolism, gastrointestinal complications.
Li, 2014 [22]	China	Orthopaedic: Total hip arthroplasty	600	300	47.3 ± 16.4	45.0 ± 10.2	25 (4.2)	15 (5.0)	90 (15.0)	35 (11.7)	Acute kidney injury
Nakhla, 2017 [23]	USA	Orthoapeadic; Lumbar fusion	263,757	239	55 ± 14	61 ± 10	–	–	35,607 (13.5)	118 (31.4)	Surgical site infection, Acute kidney injury
Sharma, 2013 [24]	Australia	Cardiac; CABG & Valve	104	30	56.5 ± 11.1	55.7 ± 11.5	20 (19.2)	6 (20.0)	25 (24.0)	9 (30.0)	In-hospital mortality, Cardiac failure, Pneumonia, Sepsis, Stroke, Return to theatre, Acute kidney injury
Stewart, 2012 [25]	USA	General; colorectal	162,986	3765	64 ± 12.1	57.4 ± 9.4	11,734 (12.3)	196 (9.2)	24,448 (25.6)	489 (22.9)	In-hospital mortality, Cardiac failure, Surgical site infection, Pneumonia, Sepsis, GI, Acute kidney Injury
Sun, 2018 [26]	USA	Urology; Penile Prosthesis	26	26	56.4 ± 9.0	53.7 ± 8.1	8 (30.8)	15 (57.7)	14 (53.9)	22 (84.6)	Return to theatre
Vargo, 2015 [27]	USA	Coronary artery bypass graft	1,705,949	2712	65.5 ± 11.8	58.2 ± 10.5	527,337 (30.9)	642 (23.7)	452,483 (26.5)	667 (24.6)	In-hospital mortality, Surgical site infection, Pneumonia, stroke, GI, Acute kidney injury

Six of the 14 studies reported outcomes solely for kidney transplant recipients [4, 16, 19, 21, 23, 25], while the remaining eight studies presented outcomes collectively for all solid organ transplants [5, 17, 18, 20, 22, 24, 26, 27]. Six studies defined clinical outcomes using the International Classification of Disease Coding (ICD) [4, 16, 19, 23, 25, 27] whilst the others reviewed medical charts without explicitly stated outcome definitions [5, 17, 18, 20–22, 24, 26]. Two studies used the Acute Kidney Injury Network (AKIN) to define AKI [17, 22]. Only 2 studies recorded graft rejection as an outcome [18, 27].

All studies reported age and gender. Ten of the 14 studies reported baseline comorbidities including ischemic heart disease and diabetes mellitus [4, 5, 17, 18, 20, 22–25, 27]. However, only 4 studies reported kidney transplant function preoperatively [5, 18, 20, 24]. No studies differentiated outcomes based on living versus deceased donor kidney status, kidney graft number or time since transplantation. Neither the aetiology of end-stage kidney disease nor the immunosuppression regimen was reported to allow stratification of outcomes based on immunosuppression doses and protocols.

### Risk of bias assessment

As per the NOS, cohort selection was of good quality, but comparability was poor due to lack of multivariable adjustment in only 5 of the 14 studies. In addition, outcome reporting was also poor due to selective reporting of and differences in outcome definitions (see Supplementary Table 2). There was no significant evidence of publication bias, as determined by funnel plot (Supplementary Figure 3a and b) and Egger’s test (*p* = 0.31). Inter-rater agreement between the two independent reviewers was very strong (κ = 0.83).

### Meta-analysis of all-cause mortality

**Fig. 2:**
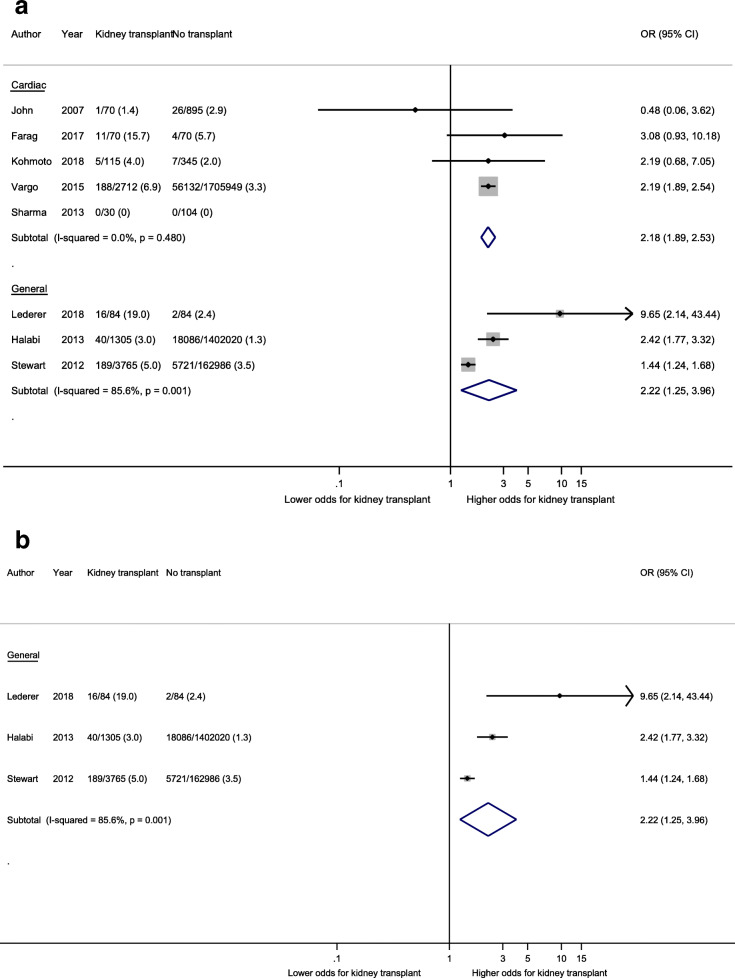
**a:** Unadjusted mortality odds of post-operative mortality in kidney transplant patients compared to non-transplant patients. **b:** Unadjusted mortality odds of peri-operative mortality in kidney transplant patients compared to non-transplant patients (Sensitivity analysis restricted to studies solely including kidney transplant patients)

The incidence of all-cause mortality ranged between 0 and 16% in kidney transplant recipients, and between 0 and 5.7% in non-transplanted patients. Eight studies involving 8151 transplant recipients reported unadjusted mortality odds ratios (Fig. 2a). Compared to their non-transplanted counterparts, the odds of mortality were more than 2-fold higher in kidney transplant recipients following cardiac surgery (5 studies, 2997 transplant recipients, OR 2.18, 95% CI 1.88–2.53, I^2^ = 0.0% p for heterogeneity = 0.48, t = 11.6, low certainty evidence), and similar following general surgery (3 studies, 5154 transplant recipients, OR 2.22 95%CI 1.25–3.96, I^2^ = 85.6% p for heterogeneity = 0.001, t = 1.9, very low certainty evidence). Fatal outcomes were not reported by any of the studies in the vascular or urological/gynaecological surgical disciplines. A single study of orthopaedic procedures reported an adjusted mortality risk ratio but did not provide absolute event rates (3209 transplant recipients, OR 2.04, 95% CI 0.96–4.33, *p* = 0.06) [16]. Sensitivity analyses including only studies of kidney transplant populations revealed similar odds using unadjusted data (3 studies in general surgery, 5154 kidney transplant recipients, OR 2.22, 95% CI 1.25–3.96, I^2^ 85.6%, p for heterogeneity = 0.001, t = 1.9, Fig. 2b). Two general surgery studies reported OR for mortality after multivariable adjustment. The mortality odds was similar to non-transplanted patients (2 studies, 5070 transplant recipients, OR 1.26, 95% CI 0.76–1.77, I^2^ 0%, p for heterogeneity = 0.75, t = 3.0). Meta-analysis results using the HKSJ method were very similar to those from the DL method. Results are summarised in Supplementary Table 3.

### Meta-analysis of morbidity outcomes

Reporting of non-fatal complications was inconsistent across studies: acute kidney injury was the most frequently reported (11 studies), followed by pneumonia (8 studies), and stroke (7 studies). Thromboembolic complications were the least frequently reported (3 studies) (Fig. 1). None of the studies graded post-operative complications by the Clavien-Dindo classification. Table 2 provides a summary of non-fatal outcomes and grading of the certainty of evidence using GRADE. Meta-analysis results using the HKSJ method were similar to those from the DL method. Results are summarised in Supplementary Table 3.

**Table 2:** Post-operative fatal and non-fatal outcomes in kidney transplant patients compared to non-transplant patients and grading of the certainty of evidence using GRADE

Outcomes (Number of Studies)	Events in kidney transplant patients	Events in non-transplanted patients	Certainty of evidence (GRADE)	Rationale					
				Risk of bias	Inconsistency	Indirectness	Imprecision	Publication bias	Strong association
30 day/In-hospital mortality (8)	449/8151	79,978/3,272,453	Low ^a,d,g,f^	↓	–	–	–	–	↑
Myocardial infarction (4)	104/4793	55,029/3,088,295	Very low ^a,b,c,g^	↓	↓↓	–	↓↓	–	–
Stroke (7)	91/7636	54,508/4,794,679	Very low ^a,b,c,g^	↓	↓↓	–	↓↓	–	–
Cardiac failure (3)	1109/7129	126,004/1,848,386	Very low ^a,c,g^	↓	↓↓	–	–	–	–
Surgical site infection (8)	293/8360	85,747/3,536,106	Very low ^a,b,c,g^	↓	↓↓	–	↓	–	–
Pneumonia (8)	693/8151	196,982/3,272,453	Very low ^a,c,g^	↓	↓↓	–	–	–	–
Sepsis (5)	692/4019	15,951/164,139	Low ^a,c,g,f^	↓	–	–	–	–	↑
Gastrointestinal events (6)	495/8006	414,890/3,272,004	Very low^a,c,g,h^	↓	↓↓	–	↓	–	–
Blood transfusion (5)	2056/7505	424,174/3,087,470	Very low^a,c,g,h^	↓	↓	–	–	–	–
Unplanned return to theatre (6)	53/395	75/1524	Very low^a,b,g^	↓	↓	–	↓↓	–	–
AKI (11)	2205/15,371	316,667/7,807,595	Moderate ^a,c,e,g^	↓	↓↓	–	–	–	↑↑
PE/DVT (3)	204/4723	81,226/3,087,400	Very low ^a,b,c,g^	↓	↓↓	–	↓↓	–	–

^a^Certainty of evidence downgraded due to concerns of poor comparability between study cohorts

^b^Certainty of evidence downgraded for imprecision, due to few events and wide confidence intervals

^c^Certainty of evidence downgraded for inconsistency due to significant heterogeneity that remained unexplained (ranged between 67% and 98%)

^d^Certainty of evidence not downgraded due to significant heterogeneity being explained by surgical disciplines and higher prevalence of diabetes in kidney transplant patients

^e^Certainty of evidence upgraded due to large (>5 fold) risk estimate

^f^Certainty of evidence upgraded due to large (>2 fold) risk estimate

^g^ Publication bias not judged to assess certainty of evidence due to small study numbers (<10)

^h^Certainty of evidence not downgraded for imprecision due to significant number of events in both groups

### Cardiovascular events (stroke and myocardial infarction)

Across all surgical types, the incidences of postoperative stroke in kidney transplant recipients ranged from 0 to 4.3%, and 0.9 to 4.0% for myocardial infarction. In non-transplanted patients the incidence of stroke and myocardial infarction ranged from 0.1 to 4.8% and 1.2 to 2.0%, respectively. The odds of stroke in kidney transplant recipients following cardiac surgery was similar to the general population (5 studies, 2997 transplant recipients, OR 1.36 95% CI 0.51–3.66, I^2^ = 53.5%, p for heterogeneity = 0.072, t- = 0.6, very low certainty evidence; Supplementary Figure 4). Meta-analysis in other surgical disciplines was not possible due to insufficient studies. Meta-analysis for postoperative myocardial infarction risk by surgical discipline and sensitivity analysis were also not possible due to insufficient studies.

### Infectious complications (pneumonia, surgical site infections, sepsis)

Five of the eight studies that reported pneumonia involved cardiac surgery where the incidence of postoperative pneumonia ranged between 1.4 to 7.1% in kidney transplant recipients and 3.8 to 7.1% in their non-transplanted counterparts. The unadjusted odds of postoperative pneumonia following cardiac surgery was similar between the two cohorts (5 studies, 2997 transplant recipients, OR 1.10 95%CI 0.95–1.28, I^2^ = 0% p for heterogeneity = 0.573, t = 1.5, very low certainty evidence, Supplementary Figure 5). Sensitivity analysis of kidney only transplant recipients revealed similar odds (3 studies in general surgery, 5154 kidney transplant recipients, OR 0.94 95% CI 0.28–3.22, I^2^ = 95.9% p for heterogeneity < 0.001, t = 0.1, very low certainty evidence). Pooling of adjusted analyses was not possible due to the fact that only one study in each of the other three disciplines reported adjusted analysis. The odds of surgical site infections was also not significantly different, irrespective of surgical discipline (4 cardiac studies, 2967 transplant recipients, OR 1.08 95% CI 0.79–1.47, I^2^ = 0.0% p for heterogeneity = 0.805, t = 0.9, very low certainty evidence and 3 general surgery studies, 5154 transplant recipients, OR 1.43 95% CI 1.03–1.98, I^2^ = 75.7% p for heterogeneity 0.016, t = 1.4, very low certainty evidence, Supplementary Figure 6).

Five studies reported sepsis as an outcome. The incidence of postoperative sepsis ranged from 2.9 to 35.7% in kidney transplant recipients, and between 1.7 to 14.3% in non-transplanted patients. The odds of sepsis was increased more than 3-fold for kidney transplant recipients following cardiac surgery (3 studies, 170 transplant recipients, OR 3.14 95% CI 1.68–5.85, I^2^ = 0% p for heterogeneity = 0.665, t = 5.7, low certainty evidence, supplementary figure 7), but comparisons by other surgical disciplines were not possible due to small study numbers.

### Acute kidney injury (AKI)

**Fig. 3:**
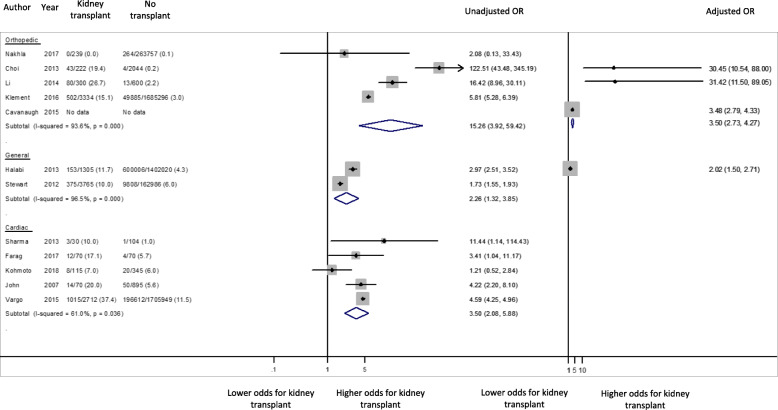
Unadjusted and adjusted AKI odds for kidney transplant patients compared to patients with non-transplant patients

The incidences of postoperative AKI in kidney transplant recipients ranged between 7.0 to 37.4% following cardiac surgery, 10.0 and 11.7% after general surgery, and 0 to 26.7% after orthopaedic surgery. The odds of postoperative AKI was higher in kidney transplant patients than in their non-transplanted counterparts (Fig. 3). The odds was highest following orthopaedic surgery (4 studies, 4095 transplant recipients, OR 15.26 95% CI 3.92–59.42, I^2^ = 93.6% p for heterogeneity< 0.001, t = 3.4, moderate certainty evidence) followed by cardiac surgery (5 studies, 2997 transplant recipients, OR 3.50 95% CI 2.08–5.88, I^2^ = 61.0% p for heterogeneity = 0.036, t = 4.4, low certainty evidence). With the exception of two studies [20, 23], the lower bound of the 95% confidence interval was greater than 1.0 in all other individual studies. Adjusted odds estimates, with age as a minimum covariate, were reported by 3 orthopaedic studies (OR 3.50 95% CI 2.73–4.27, I^2^ = 48% *p* = 0.146, t = 12.8) and a single study involving general surgery (OR 2.02 95% CI 1.42–2.63).

Other morbidity outcomes, including urinary tract infections, return to theatre, transfusion requirements and thromboembolic events, are summarised in Supplementary Table 3.

### Heterogeneity

#### Mortality

The heterogeneity in the pooled estimate of the unadjusted mortality odds ratio varied from 0% in studies involving cardiac surgery to 85.6% in general surgery. Heterogeneity was significantly reduced in the adjusted odds estimate (I^2^ 0%, *p* = 0.65). L’Abbe plots did not identify any studies that exerted disproportionate influence on derived estimates of effect (Supplementary Figure 1). Weighted univariable meta-regression analyses of study-level characteristics demonstrated a significant association between postoperative mortality and prevalence of diabetes mellitus (slope 0.03, *p* = 0.041; Supplementary Figure 8), but not with the presence of ischaemic heart disease. No significant association was observed between postoperative mortality and other study characteristics, such as single or multiple centre design, or methodological study quality as assessed by the Newcastle Ottawa Scale.

#### Morbidity

L’Abbe plots did not identify study cohorts that may have explained the observed results (Supplementary Figure 2). Weighted univariable meta-regression to explore heterogeneity of postoperative pneumonia and surgical site infection odds estimates were not possible due to insufficient study numbers. Meta-regression of AKI odds and study-level patient characteristics, including age, presence of diabetes mellitus and ischemic heart disease, did not explain the observed difference. The influence of other important factors, such as the use of calcineurin inhibitors, deceased versus living donor kidney transplant, time since transplantation, and baseline kidney function, could not be evaluated as this information was not made available to the authors.

## Discussion

This systematic review and meta-analysis of 14 studies involving 15,481 transplant recipients and 7,807,705 non-transplanted patients identified that kidney transplant recipients have an increased odds of postoperative death following elective cardiac and general surgery, especially in the setting of diabetes, and a substantially increased odds of acute kidney injury. These findings extend those of previous studies reporting increased risks of postoperative mortality in individuals with non-dialysis and dialysis-requiring chronic kidney disease [28, 29]. Thus, the presence of a kidney transplant is an important consideration when discussing peri-operative risk with patients.

Whilst this review consistently demonstrated that kidney transplant recipients were at increased odds of postoperative mortality, the magnitude and cause of this heightened odds remains uncertain. Meta-regression demonstrated that the excess postoperative mortality odds attributable to having a kidney transplant was further increased by the presence of diabetes mellitus, which is an established independent risk factor for post-operative mortality [30]. This finding is of importance given the increasing prevalence of obesity in the kidney transplant recipient population and its association with the development of type 2 diabetes mellitus [31]. The reasons for a heightened postoperative mortality odds in kidney transplant recipients could potentially be related to the adverse immunologic, cardiovascular and metabolic effects of immunosuppression. However, data on cause of death were insufficient to explore mechanisms of heightened risk. Cause of death was only reported in a single study in which kidney transplant recipients who developed an infectious complication had a higher mortality odds than those who did not (OR 2.79; 95% CI 1.93–4.04) [25]. Whilst studies signalled an increased odds of postoperative sepsis, the odds of other infective complications, including surgical site infection and pneumonia, were not appreciably different to those of the general population. This may be explained by the limited reporting of these postoperative complications in cohort studies and the lack of standardised definitions.

Similar findings were observed for postoperative cardiovascular outcomes. Specifically, the odds of postoperative stroke in kidney transplant recipients was no higher than that of non-transplanted patients. This observation is somewhat surprising given the fact that the risk of premature cardiovascular disease after kidney transplantation is reported in observational cohort studies to be three to five times that of the general population [32]. This apparent discrepancy in findings may be explained by selective outcome reporting and highlights the need for accurate reporting of patient comorbidities, preoperative kidney function, kidney disease aetiology, immunosuppression regimen, cause of death and outcomes according to standard definitions in studies of kidney transplant recipients undergoing surgery. Nevertheless, current transplant guidelines recommend screening for underlying coronary artery disease prior to activation on the transplant wait list [33]. However, there are no such recommendations when kidney transplant recipients are considering elective surgery and clinicians need to consider assessing risk based on functional capacity as recommended by the American College of Cardiology/American Heart Association (ACC/AHA) [34].

The other key finding of this review was that the odds of post-operative acute kidney injury in kidney transplant recipients was considerably higher than in non-transplanted patients in both unadjusted and sensitivity analyses. A single study reported a higher incidence of postoperative serum creatinine elevation in kidney transplant recipients with a preoperative serum creatinine greater than 176 μmol/L compared to those with lower values (47.6% vs. 18.2%, *p* = 0.04) [5]. In the general population, a higher preoperative serum creatinine is associated with an increased risk of developing a perioperative acute kidney injury [35]. In the absence of access to patient-level data, adjustment for baseline kidney transplant function was not possible across the studies. Furthermore, it is unclear what proportion of these events was related to graft rejection, and if the injury was severe enough to require temporary dialysis. Future studies are needed to confirm these findings and the long-term impact of postoperative acute kidney injury to better facilitate informed decision making.

Although a comprehensive search strategy and rigorous assessment of methodologic quality using a validated tool were used, there were limitations which contributed to the low certainty of evidence. Firstly, observational studies and their subsequent meta-analyses are prone to bias due to the inability to quantify and adjust for known and unknown confounders. This was compounded by the incomplete reporting of patient comorbidities, preoperative kidney function, precise immunosuppressive regimens, selective reporting of clinical outcomes, and variation of outcome definitions between studies. In addition, assessment of publication bias using the funnel plot was limited by small number of studies [36]. Meta-analysis of adjusted odds risks may have overcome some of the limitations mentioned but these were poorly reported. Secondly, selective reporting of non-fatal complications and lack of standardised classification of complications using the Clavien-Dindo did not allow fair comparison of outcomes between surgical types. Thirdly, 5% of the solid organ recipients were comprised of non-kidney transplant recipients but this was necessary to avoid excluding a number of key studies. Fourthly, there were no studies available for inclusion involving potentially high-risk surgical procedures such as elective vascular surgery. Finally, as this study excluded patients having emergent surgery, these results should not be extrapolated beyond informing elective surgery risks.

## Conclusion

Patients with a kidney transplant who undergo elective surgery are at increased odds of postoperative mortality compared to non-transplanted patients, with higher odds among patients with diabetes mellitus. Kidney transplant recipients are also at increased odds of postoperative acute kidney injury, but the odds of postoperative cardiovascular complications was no different between the two groups. The findings of this review also highlight the need for further studies to be more comprehensive in reporting patient baseline characteristics, including graft function and immunosuppression, as well as the use of consistent outcome definitions and recording of both short- and long-term graft outcomes. This will inform decision-making around the appropriateness of elective surgery in kidney transplant recipients and allow clinicians to adopt means to mitigate perioperative risk.

## Supplementary information

**Additional file 1 **: **Supplementary Table 1a:** Search strategy for EMBASE. **Supplementary Table 1b:** Search strategy for MEDLINE. **Supplementary Table 2:** Methodological quality of each study assessed by the NOS scale. **Supplementary Figure 1:** L’Abbe plot for mortality event rates. **Supplementary Figure 2:** L’Abbe plot for post-operative AKI event rates. **Supplementary Figure 3a:** Funnel plot for bias assessment (mortality). **Supplementary Figure 3b:** Funnel plot for bias assessment (Acute kidney injury). **Supplementary Table 3:** Meta-analysis results using HKSJ method. **Supplementary Figure 4:** Unadjusted odds ratio estimate of post-operative stroke in kidney transplant patients compared to patients with non-transplant patients. **Supplementary Figure 5:** Unadjusted odds ratio estimate of post-operative pneumonia in kidney transplant patients compared to non-transplant patients. **Supplementary Figure 6:** Unadjusted odds ratio estimate of surgical site infection in kidney transplant patients compared to non-transplant patients. **Supplementary Figure 7:** Unadjusted odds ratio estimate of sepsis in kidney transplant patients compared to patients with non-transplant patients. **Supplementary Figure 8:** Meta-regression for post-operative mortality odds by prevalence of diabetes mellitus. Every circle represents a study; the circle size is representative of the weight of that study in the analysis. The relation between logarithmic mortality odds ratio and the prevalence of diabetes mellitus in kidney transplant patients and non-transplanted patients is significant (slope 0.03, 95% CI 0.01–0.05, *I2* 19.2%, adjusted R^2^ 100%, *p* = 0.041). **Supplementary Table 3**: Meta-analysis of other non-fatal post-operative complications.

## Data Availability

Data uploaded as an additional file. No individual patient data was used.

## Abbreviations

AKI: Acute kidney injury
CI: Confidence intervals
ESKD: End-stage kidney disease
GRADE: Grading of Recommendations, Assessment, Development and Evaluations
ICD: International classification of disease
NOS: Newcastle-Ottawa Scale
OR: Odds Ratio
