# Supplementary material for: Postoperative outcomes of kidney transplant recipients undergoing non-transplant-related elective surgery: a systematic review and meta-analysis
Source: BMC Nephrol. 2020 Aug 25;21:365. doi: 10.1186/s12882-020-01978-4 (PMC7448361; doi:10.1186/s12882-020-01978-4)
Supplement: Supplementary file 1 — Additional file 1 : Supplementary Table 1a: Search strategy for EMBASE. Supplementary Table 1b: Search strategy for MEDLINE. Supplementary Table 2: Methodological quality of each study assessed by the NOS scale. Supplementary Figure 1: L’Abbe plot for mortality event rates. Supplementary Figure 2: L’Abbe plot for post-operative AKI event rates. Supplementary Figure 3a: Funnel plot for bias assessment (mortality). Supplementary Figure 3b: Funnel plot for bias assessment (Acute kidney injury). Supplementary Table 3: Meta-analysis results using HKSJ method. Supplementary Figure 4: Unadjusted odds ratio estimate of post-operative stroke in kidney transplant patients compared to patients with non-transplant patients. Supplementary Figure 5: Unadjusted odds ratio estimate of post-operative pneumonia in kidney transplant patients compared to non-transplant patients. Supplementary Figure 6: Unadjusted odds ratio estimate of surgical site infection in kidney transplant patients compared to non-transplant patients. Supplementary Figure 7: Unadjusted odds ratio estimate of sepsis in kidney transplant patients compared to patients with non-transplant patients. Supplementary Figure 8: Meta-regression for post-operative mortality odds by prevalence of diabetes mellitus. Every circle represents a study; the circle size is representative of the weight of that study in the analysis. The relation between logarithmic mortality odds ratio and the prevalence of diabetes mellitus in kidney transplant patients and non-transplanted patients is significant (slope 0.03, 95% CI 0.01–0.05, I2 19.2%, adjusted R2 100%, p = 0.041). Supplementary Table 3: Meta-analysis of other non-fatal post-operative complications. [file 12882_2020_1978_MOESM1_ESM.docx]

# Supplementary material:

The EMBASE search strategy is shown below (Table 1a) and will be used to identify possibly relevant papers. Similarly, the MEDLINE search strategy is in Table 1b.

**Table 1a:** Search strategy for EMBASE.

**POPULATION:**

'kidney transplantation':de,ti,ab OR 'kidney transplant':de,ti,ab OR 'kidney allograft':de,ti,ab OR 'renal transplantation':de,ti,ab OR 'renal transplant':de,ti,ab OR 'renal allograft':de,ti,ab

**EXPOSURE:**

surgery:de,ti,ab

**OUTCOMES:**

'postoperative complication'/mj OR 'cardiovascular mortality'/exp OR 'hospital mortality'/exp OR 'surgical mortality'/exp OR 'anastomosis dehiscence'/exp OR 'anastomosis leakage'/exp OR 'failed back surgery syndrome'/exp OR 'gastric band erosion'/exp OR 'vein graft disease'/exp OR 'paravalvular leak'/exp OR 'postoperative edema'/exp OR 'postoperative hemorrhage'/exp OR 'postoperative ileus'/exp OR 'postoperative infection'/exp OR 'postoperative inflammation'/exp OR 'postoperative thrombosis'/exp OR 'periprosthetic fracture'/exp OR 'prosthetic valve dysfunction'/exp OR 'surgical infection'/exp OR 'surgical injury'/exp OR 'heart infarction'/exp OR 'cerebrovascular accident'/exp OR 'hospital readmission'/exp OR 'reoperation'/exp OR 'blood transfusion' OR 'acute kidney injury'/exp OR 'urinary tract infection'/exp

**Table 1b:** Search strategy for MEDLINE.

**POPULATION**

AB kidney transplantation OR TI kidney transplantation OR AB kidney transplant OR TI kidney transplant OR AB renal transplantation OR TI renal transplantation OR AB renal transplant OR TI renal transplant OR AB kidney allograft OR TI kidney allograft OR AB renal allograft OR TI renal allograft

**EXPOSURE:**

(AB surgery OR TI surgery) OR MM "Specialties, Surgical+"

**OUTCOMES:**

(MM “Postoperative Complications”) OR (MH "Cause of Death") OR (MH "Survival") OR (MH "Arrhythmias, Cardiac+") OR (MH "Death+") OR (MH "Hemorrhage+") OR (MH "Hypovolemia") OR (MH "Seroma") OR (MH "Serositis") OR (MH "Abscess") OR (MH "Cellulitis") OR (MH "Empyema") OR (MH "Systemic Inflammatory Response Syndrome+") OR (MH "Intraoperative Complications") OR (MH "Infarction") OR (MH "Fat Necrosis") OR (MH "Gangrene") OR (MH "Femur Head Necrosis") OR (MH "Anastomotic Leak") OR (MH "Graft Occlusion, Vascular") OR (MH "Incisional Hernia") OR (MH "Malignant Hyperthermia") OR (MH "Postcholecystectomy Syndrome") OR (MH "Postgastrectomy Syndromes") OR (MH "Postoperative Hemorrhage") OR (MH "Postpericardiotomy Syndrome") OR (MH "Prosthesis-Related Infections") OR (MH "Prosthesis Failure") OR (MH "Surgical Wound Dehiscence") OR (MH "Surgical Wound Infection") OR (MH "Respiratory Aspiration+") OR (MH "Multiple Organ Failure") OR (MH "Shock, Cardiogenic") OR (MH "Shock, Hemorrhagic") OR (MH "Ulcer") OR (MH "Arrhythmias, Cardiac") OR (MH "Death, Sudden, Cardiac") OR (MH "Out-of-Hospital Cardiac Arrest") OR (MH "Edema, Cardiac") OR (MH "Heart Failure, Diastolic") OR (MH "Heart Failure, Systolic") OR (MH "Acute Coronary Syndrome") OR (MH "Angina, Unstable+") OR (MH "Coronary Artery Disease") OR (MH "Coronary Occlusion") OR (MH "Anterior Wall Myocardial Infarction") OR (MH "Inferior Wall Myocardial Infarction") OR (MH "Non-ST Elevated Myocardial Infarction") OR (MH "Shock, Cardiogenic") OR (MH "ST Elevation Myocardial Infarction") OR (MH "Coronary Thrombosis") OR (MH "Postpericardiotomy Syndrome") OR (MH "Cerebral Infarction") OR (MH "Stroke, Lacunar") OR (MH "Brain Infarction") OR (MH "Bronchopneumonia") OR (MH "Pneumonia, Aspiration") OR (MH "Pneumonia, Bacterial") OR (MH "Pulmonary Atelectasis") OR (MH "Pulmonary Edema") OR (MH "Pulmonary Embolism") OR (MH "Length of Stay") OR (MH "Patient Readmission") OR (MH "Erythrocyte Transfusion") OR (MH "Blood Transfusion") OR (MH "Reoperation") OR (MH "Second-Look Surgery") OR (MH "acute kidney injury")OR (MH "urine tract infection")

**Supplementary Table 2:** Methodological quality of each study assessed by the NOS scale.

| **First Author, Year** | **Cohort Study Design** | **Selection** /**🟑🟑🟑🟑** | **Comparability** /**🟑🟑** | **Outcomes** /**🟑🟑🟑** |
| --- | --- | --- | --- | --- |
| Cavanaugh, 2015 | Retrospective | **🟑🟑🟑🟑** | **🟑🟑** | **🟑🟑🟑** |
| Choi, 2013 | Retrospective | **🟑🟑🟑** | **-** | **🟑🟑🟑** |
| Farag, 2017 | Retrospective | **🟑🟑🟑** | **🟑🟑** | **🟑🟑🟑** |
| Halabi, 2013 | Retrospective | **🟑🟑🟑🟑** | **🟑🟑** | **🟑🟑🟑** |
| John, 2007 | Retrospective | **🟑🟑🟑** | - | **🟑🟑🟑** |
| Klement, 2016 | Retrospective | **🟑🟑🟑🟑** | - | **🟑🟑🟑** |
| Kohmoto 2018 | Retrospective | **🟑🟑🟑🟑** | - | **🟑🟑🟑** |
| Lederer 2018 | Retrospective | **🟑🟑** | - | **🟑🟑** |
| Li, 2014 | Retrospective | **🟑🟑🟑** | **🟑🟑** | **🟑🟑🟑** |
| Nakhla, 2017 | Retrospective | **🟑🟑🟑** | - | **🟑🟑** |
| Sharma, 2013 | Retrospective | **🟑🟑🟑** | - | **🟑🟑🟑** |
| Stewart, 2012 | Retrospective | **🟑🟑🟑** | **🟑🟑** | **🟑🟑🟑** |
| Sun 2018 | Retrospective | **🟑🟑🟑🟑** | - | **🟑🟑🟑** |
| Vargo, 2015 | Retrospective | **🟑🟑🟑** | - | **🟑🟑🟑** |

**Selection criteria (out of 4 stars):** Representation of exposed cohort, selection of non-exposed group, ascertainment of exposure, demonstration that outcome of interest was not present at start of study

**Comparability (out of 2 stars):** Comparability of cohorts by the design or analysis

**Outcomes (out of 3 stars):** Assessment of outcomes, Follow-up duration and adequacy of follow-up of cohorts

**Supplementary Figure 1:** L’Abbe plot for mortality event rates.

**Supplementary Figure 2:** L’Abbe plot for post-operative AKI event rates.

**Supplementary Figure 3a:** Funnel plot for bias assessment (mortality)

**Supplementary Figure 3b:** Funnel plot for bias assessment (Acute kidney injury)

**Supplementary Table 3: Meta-analysis results using HKSJ method**

| **Outcome (number of studies)** | **OR** | **95%CI** | **P (2-tailed)** | **t-statistic** |
| --- | --- | --- | --- | --- |
| **Mortality** |  |  |  |  |
| Cardiac (4) | 2.18 | (1.98-2.36) | 0.001 | 11.6 |
| General (3) | 2.22 | (1.22-4.02) | 0.189 | 1.9 |
| General (Adjusted) (2) | 1.26 | (1.03-1.53) | 0.203 | 3.0 |
| **Stroke** |  |  |  |  |
| Cardiac (5) | 1.36 | (0.83 – 2.24) | 0.563 | 0.6 |
| **Pneumonia** |  |  |  |  |
| Cardiac (5) | 1.10 | (1.02-1.17) | 0.218 | 1.5 |
| General (3) | 0.94 | (0.35-2.54) | 0.935 | 0.1 |
| **SSI** |  |  |  |  |
| Cardiac (4) | 1.08 | (0.98-1.19) | 0.456 | 0.9 |
| General (3) | 1.43 | (0.99-2.06) | 0.290 | 1.4 |
| **Sepsis** |  |  |  |  |
| Cardiac | 3.14 | (2.34-4.21) | 0.029 | 5.7 |
| **Acute kidney Injury** |  |  |  |  |
| Orthopaedics (4) | 15.26 | (5.97-38.86) | 0.044 | 3.4 |
| Cardiac (5) | 3.50 | (2.62-4.66) | 0.011 | 4.4 |
| Orthopaedic (Adjusted) (3) | 3.50 | (2.76-3.58) | 0.006 | 12.8 |
| **Return to theatre** |  |  |  |  |
| Cardiac (4) | 1.66 | (0.94-2.94) | 0.380 | 1.0 |
| **Urinary Tract Infection** |  |  |  |  |
| General (2) | 2.34 | (0.78-7.02) | 0.299 | 2.0 |
| **Transfusion requirement** |  |  |  |  |
| Cardiac (2) | 2.95 | (0.31-27.66) | 0.435 | 1.2 |
| General (2) | 1.74 | (0.01-396.62) | 0.838 | 0.3 |
| **Thromboembolic events** |  |  |  |  |
| General (2) | 0.62 | (0.01-25.79) | 0.799 | 0.3 |

**Supplementary Figure 4:** Unadjusted odds ratio estimate of post-operative stroke in kidney transplant patients compared to patients with non-transplant patients.

**Supplementary Figure 5:** Unadjusted odds ratio estimate of post-operative pneumonia in kidney transplant patients compared to non-transplant patients.

**Supplementary Figure 6:** Unadjusted odds ratio estimate of surgical site infection in kidney transplant patients compared to non-transplant patients.

**Supplementary Figure 7:** Unadjusted odds ratio estimate of sepsis in kidney transplant patients compared to patients with non-transplant patients

**Supplementary Figure 8:** Meta-regression for post-operative mortality odds by prevalence of diabetes mellitus. Every circle represents a study; the circle size is representative of the weight of that study in the analysis. The relation between logarithmic mortality odds ratio and the prevalence of diabetes mellitus in kidney transplant patients and non-transplanted patients is significant (slope 0.03, 95% CI 0.01 – 0.05, *I2* 19.2%, adjusted R^2^ 100%, p=0.041).

**Supplementary Table 3: Meta-analysis of non-fatal post-operative complications**

|  | **Unadjusted odds ratio** | | | | | | | **Adjusted odds ratios** | | | |
| --- | --- | --- | --- | --- | --- | --- | --- | --- | --- | --- | --- |
| **Outcome** | **Type of Surgery** | **Number of studies** | **Transplant patients** | **Non-transplant patients** | **Overall effect OR (95% CI) ^c^** | ***I*^2^(%)** | **P** | **Number of studies** | **Overall effect OR (95% CI)^c^** | ***I*^2^** | **P** |
| Return to theatre | Cardiac | 4 | 285 | 1,414 | 1.66 (0.63-4.40) | 39.6 | 0.174 | - | - | - | - |
|  | General | 1 | 84 | 84 | 2.43 (1.22-4.84) | - | - | - | - | - | - |
|  | Orthopaedic | 0 | - | - | - | - | - | - | - | - | - |
|  | Vascular | 0 | - | - | - | - | - | - | - | - | - |
|  | Urology/gynaecology | 1 | 26 | 26 | 1.00 (0.18-5.48) | - | - | - | - | - | - |
| Urinary tract infection | Cardiac | 1 | 70 | 70 | 3.09 (0.31 – 30.45) | - | - | - | - | - | - |
|  | General | 2 | 1389 | 1,402,104 | 2.3 (1.00 – 5.48) | 39.2 | 0.200 | - | - | - | - |
|  | Orthopaedic | 0 | - | - | - | - | - | - | - | - | - |
|  | Vascular | 0 | - | - | - | - | - | - | - | - | - |
|  | Urology/gynaecology | 0 | - | - | - | - | - | - | - | - | - |
| Transfusion requirement | Cardiac | 2 | 2,782 | 1,706,019 | 2.95 (0.52-16.64) | 67.2 | 0.081 | - | - | - | - |
|  | General | 2 | 1,389 | 1,402,104 | 1.74 (0.03-115.65) | 93.9 | 0.000 | - | - | - | - |
|  | Orthopaedic | 1 | 3,334 | 1,685,296 | 1.43 (0.66-1.98) | - | - | - | - | - | - |
|  | Vascular | 0 | - | - | - | - | - | - | - | - | - |
|  | Urology/gynaecology | 0 | - | - | - | - | - | - | - | - | - |
| Thromboembolic events (DVT/PE*) | Cardiac | 0 | - | - | - | - | - | - |  |  |  |
|  | General | 2 | 1,389 | 1,402,104 | 0.62 (0.03-12.50) | 50.1 | 0.157 | - | - | - | - |
|  | Orthopaedic | 1 | 3,334 | 1,685,296 | 1.35 (0.42-3.08) | - | - | 1 | 2.07 (1.46-2.94) | - | - |
|  | Vascular | 0 | - | - | - | - |  | - | - | - | - |
|  | Urology/gynaecology | 0 | - | - | - | - | - | - | - | - | - |

*DVT/PE: Deep Venous Thrombosis/Pulmonary Embolism
